# Functional Nanostructured Lipid Carrier-Enriched Hydrogels Tailored to Repair Damaged Epidermal Barrier

**DOI:** 10.3390/gels10070466

**Published:** 2024-07-16

**Authors:** Radwan Joukhadar, Laura Nižić Nodilo, Jasmina Lovrić, Anita Hafner, Ivan Pepić, Mario Jug

**Affiliations:** Faculty of Pharmacy and Biochemistry, University of Zagreb, A. Kovačića 1, 10 000 Zagreb, Croatia; radwan.joukhadar@gmail.com (R.J.); laura.nizic@pharma.unizg.hr (L.N.N.); jasmina.lovric@pharma.unizg.hr (J.L.); anita.hafner@pharma.unizg.hr (A.H.); ivan.pepic@pharma.unizg.hr (I.P.)

**Keywords:** nanostructured lipid carriers, nanocomposite hydrogel, polysaccharides, cutaneous application, rheological properties, textural properties, biocompatibility, skin hydration

## Abstract

In this study, a functional nanostructured lipid carriers (NLCs)-based hydrogel was developed to repair the damaged epidermal skin barrier. NLCs were prepared via a high-energy approach, using argan oil and beeswax as liquid and solid lipids, respectively, and were loaded with ceramides and cholesterol at a physiologically relevant ratio, acting as structural and functional compounds. Employing a series of surfactants and optimizing the preparation conditions, NLCs of 215.5 ± 0.9 nm in size and a negative zeta potential of −42.7 ± 0.9 were obtained, showing acceptable physical and microbial stability. Solid state characterization by differential scanning calorimetry and X-ray powder diffraction revealed the formation of imperfect crystal NLC-type. The optimized NLC dispersion was loaded into the gel based on sodium hyaluronate and xanthan gum. The gels obtained presented a shear thinning and thixotropic behavior, which is suitable for dermal application. Incorporating NLCs enhanced the rheological, viscoelastic, and textural properties of the gel formed while retaining the suitable spreadability required for comfortable application and patient compliance. The NLC-loaded gel presented a noticeable occlusion effect in vitro. It provided 2.8-fold higher skin hydration levels on the ex vivo porcine ear model than the NLC-free gel, showing a potential to repair the damaged epidermal barrier and nourish the skin actively.

## 1. Introduction

The skin is an interface between us and our environment, presenting a flexible barrier that provides protection, immunity, regulation, and sensation. *Stratum corneum*, the outermost epidermal layer, is the principal permeability and protective barrier composed of corneocytes embedded into a multilamellar lipid matrix of unique composition, comprising ceramides (about 50%, *w*/*w*), free fatty acids (10–20%, *w*/*w*), and cholesterol (25%, *w*/*w*). It also contains a natural moisturizing factor, various enzymes and their inhibitors, antimicrobial peptides, and lipids, which work interactively to maintain the epidermal barrier function [1,2]. *Stratum corneum* lipids disorganization and altered epidermal barrier function are involved in several skin disorders and diseases, including dry skin and sensitive skin, as well as atopic dermatitis, psoriasis, and ichthyosis [1,2,3,4]. Topical corticosteroids and emollients have been the foundation of the therapy for decades [3,5]. However, topical corticosteroids result in significant side effects and further weakening of the skin barrier after long-term use, while emollients alone are often insufficient [5]. Therefore, there is a need to develop new topical formulations that could actively restore the impaired skin barrier by replenishing the lacking skin lipids [3,5]. 

Nanostructured lipid carriers (NLCs)-containing composite hydrogels may be suitable formulations for repairing the damaged dermal barrier. Such a system where NLCs are non-covalently immobilized in a hydrogel matrix provides superior functionality due to the synergistic action of components, enabling an enhanced physical stability of included nanocarriers, combined with the more controlled release of active ingredients and prolonged retention at the application site. Such nanocomposite hydrogels have been engineered for several biomedical applications, including drug delivery, tissue engineering, and wound healing [6,7]. We have selected sodium hyaluronate (HA) and xanthan gum (XA) as the hydrogel base, considering their biocompatibility, biodegradability, non-immunogenic, and non-toxic nature, as well as environmental compatibility [8]. The high molecular weight HA (above 1 × 10^6^ Da) forms cohesive, viscous, lubricious aqueous gels with excellent moisturizing activity on the skin, enhances wound healing, and promotes dermal permeation of nanocarriers like liposomes [8,9]. The XA was selected due to its thickening capacity, and both the pH and thermal stability of the hydrogels formed [7,8]. 

Among the numerous types of nanocarriers, NLCs have presented exceptional utility for cutaneous applications [10]. NLCs were introduced as a second generation of lipid nanoparticles with improved drug loading and stability characteristics, owing to a less organized lipid core brought up by the presence of liquid lipids that may comprise up to 30% (*w*/*w*) of the lipid matrix [10,11]. The benefits of NLC dermal application include skin protection and repair by reinforcement of the damaged epidermal lipid matrix. Furthermore, the formation of the NLC (mono)layer presents an occlusive film that prevents water loss by evaporation, thus enhancing skin hydration and elasticity. The ability to block UV rays by light scattering makes NLCs an efficient sunscreen. Finally, the spherical shape of NLCs provides them with exceptional lubrication, reducing the friction between the skin and clothing or between the adjacent skin areas [10]. By careful selection of the lipids and surfactants, it is possible to further enhance the emollient properties of NLCs while maintaining their high biocompatibility [11,12,13]. 

In the development of NLCs, we focused the interest on natural compounds, in line with consumer preferences and growing environmental concerns [14]. As a liquid lipid, we have selected argan oil (AO), showing a similar content of glycerol esters as they appear in the human sebum, preventing skin dehydration, supporting the treatment of skin pimples, scars, wrinkles, and protecting the skin from UV-B radiation [15]. Beeswax (BW) was selected as a naturally occurring bee product, acting as an occlusive by creating a semi-occlusive skin barrier that minimizes transepidermal water loss and locks in hydration. BW is an emollient able to soften and soothe the skin and has been shown to help alleviate symptoms associated with skin conditions like dermatitis and psoriasis and overgrowth of normal skin flora [16]. Ceramide NG (CE) and cholesterol (CH) were selected as both structural and functional lipids and were loaded into the NLCs at a physiological 2/1 weight ratio, which is in line with their essential role in the formation of the epidermal barrier [1,4,5,17]. 

The selection of surfactants is crucial for NLC formation for a targeted size range for skin application (150–300 nm) and their colloidal stability and biocompatibility. We have opted for polysorbates and poloxamers, as they are among the most frequently used surfactants for NLC preparation. Polysorbates are used in many marketed products for topical and dermal application, while poloxamers are recommended for use in dermal and oral liquid and semisolid formulations [11,13]. However, as PEG-contained surfactants may impair the skin barrier, we have also considered sucrose esters (sucrose monostearate (SS) and sucrose monopalmitate (SM)) as biodegradable non-ionic surfactants, which are generally better tolerated and less irritating than polyethoxylated surfactants [18]. An α-tocopheryl acetate (αTA) was used to chemically stabilize the lipids employed in NLC formulation, while pentylene glycol (PG) was used as a preservative to ensure the antimicrobial stability of the formulations, considering its good skin tolerability, suitability for NLC-based formulations and other technological and cosmetic benefits, like acting as a solvent, humectant, and texture modifier for the skin formulations [19,20]. 

The NLC dispersions were prepared by applying a high-energy approach [13]. The first part of the study aimed to select the adequate emulsifier for the preselected combination of liquid and solid lipids, resulting in particles of the targeted size range and adequate colloidal stability. The prepared NLC dispersions’ physical stability was analyzed using accelerated and real-time conditions, while the antimicrobial stability was tested in line with the Pharmacopeial requirements. Differential scanning calorimetry (DSC) and X-ray powder diffraction (XRPD) were used to determine the type and structure of NLC formed. In the second part of the study, the optimized NLC dispersion was incorporated into hydrogels of adequate rheological and textural properties for dermal application. NLC dispersion and hydrogel formulation biocompatibility were tested in vitro using an immortalized human epidermal keratinocyte cell line (HaCaT). Finally, the functional properties of the NCL-loaded hydrogels were assessed using an in vitro occlusion test and ex vivo skin hydration test to validate their potential to repair the damaged dermal barrier. 

## 2. Results and Discussion

### 2.1. Preparation and Optimization of NLC Dispersion

Functional NLC were prepared using the combined high speed and ultrasonication approach. High-speed homogenization was used to mix the melted lipid phase with a pre-heated aqueous phase containing the surfactant, while the ultrasonication step was added to homogenize the system further. Although a probe sonicator is preferred over a bath sonicator, as it is more efficient, its use can cause metal contamination for the sample. The lower efficiency of the sonicator bath may be overcome by optimizing the sonication time to obtain the targeted particle size without overheating the sample [13].

**Figure 1:**
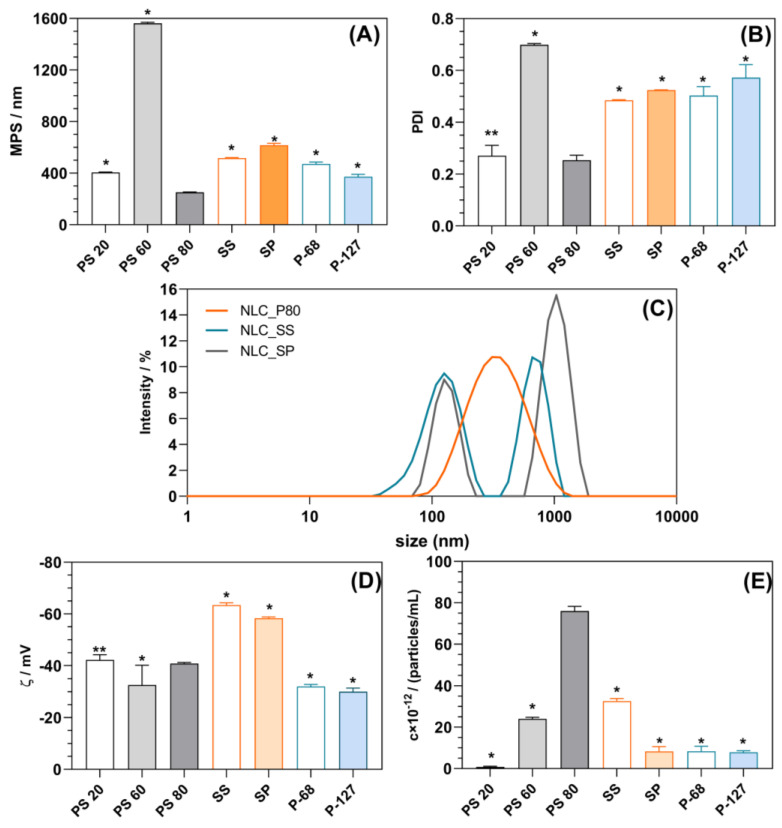
The effect of the selected emulsifier/GMS blends on the properties of NLCs formed: mean particle size (*MPS*, **A**), polydispersity index (*PDI*, **B**), particle size distribution (**C**), zeta potential (ζ, **D**), and concentration of the particles formed (**E**). One asterisk (*) denotes a statistically significant difference compared to PS80 (*p* < 0.0001), while two asterisks (**) denote a statistically insignificant difference compared to PS80 (*p* > 0.05).

In the preparation of NLC, the surfactants are used to reduce the interfacial energy between the lipid and aqueous phases during the preparation and to provide physical stability of NLC dispersion during storage [12]. Surfactants are usually selected based on the intended route of administration and hydrophile–lipophile balance (HLB) value that needs to be matched to that of the oil phase of the formulation [13]. Such an approach may be particularly challenging when using lipids of natural origin due to batch-to-batch variability in their content and when the HLB value for a substance is not readily available, like in the case of CE. Considering that, we opted to explore the suitability of different surfactants combined with glyceryl monostearate (GMS) at the same *w*/*w* ratio, owing to its confirmed synergism with other surfactants, resulting in the most stable emulsion formation [21]. In fact, obtaining an NLC of the desired lipid composition in the targeted size range without GMS was not possible. Furthermore, the usage of more than one surfactant was reported to more efficiently reduce the PDI of the NLC dispersion, as compared to that obtained by the use of only one surfactant [22]. As elaborated previously, the lipid composition of the NLC formulation was preselected based on the desired functionality, and the efficiency of the emulsifier mixture employed was evaluated based on the obtained mean particle size (MPS), polydispersity index (PDI), zeta potential, and concentration of the particles obtained (Figure 1). In this phase, the pre-emulsion was prepared at 12,000 rpm.

Among tested combinations, PS80/GMS appeared the most efficient, producing monodisperse NLC (PDI 0.25 ± 0.02) with a size of 251.90 ± 3.77 nm and a zeta potential of −40.16 ± 0.55 mV, which contributed to the NLC’s colloidal stability (Figure 1A–D). Furthermore, using PS80/GMS resulted in an NLC dispersion containing the highest concentration of the particles (Figure 1E). The combinations of GMS with other polysorbates tested (i.e., P20 and P60) were not suitable, as P60 led to the formation of polydisperse particles of unacceptable size (MPS 1562 ± 8 nm, Figure 1A,B), while PS20 resulted in NLCs with the lowest particle concentration (Figure 1E). The NLC dispersions prepared using sucrose esters were polydisperse, presenting two populations of the particles. For NLC_SS, the first peak was observed at 125.6 ± 3.0 nm, corresponding to the primary particles, and the second at 721.6 ± 36.5 nm, probably related to the aggregates of the primary particles. In the case of NLC_SP, the first peak appeared at a similar value of 131.25 ± 0.69 nm, while the second peak, corresponding to the aggregates, was observed at 986.59 ± 10.36 nm, indicating an even higher tendency toward agglomeration in this sample. Although NLC_SS and NLC_SM presented an elevated negative zeta potential compared to other samples (i.e., −63.45 ± 0.46 mV and −58.39 ± 0.46 mV, respectively; *p* < 0.0001), such an electrostatic effect was not sufficient to provide the colloidal stability of the system. Probably PS80 (i.e., polyoxyethylene (20) sorbitan monooleate) due to the presence of long polyoxyethylene chains in the structure provided an additional steric stabilization of the system despite lower zeta potential. Such functionality resulted in the fact that PS80 is the most widely used surfactant in the development of NLC formulations for dermal and topical administration [13]. 

In this light, one would expect the high suitability of poloxamers as emulsifiers (i.e., F68 and F127), owing to their triblock structure consisting of poly(ethylene oxide) (PEO) and poly(propylene oxide) (PPO) units [23]. However, they were less efficient than PS80, resulting in polydisperse NLC samples with a mean particle size outside the targeted range. In general, associative interactions with subsequent tight molecular packaging are usually observed between non-ionic surfactants containing fatty acids as hydrophobic parts and lipids, thus forming smaller particles. In the case of P-60 and P-127, the interaction of PPO moiety and lipids employed was not preferable, resulting in larger particles.

**Figure 2:**
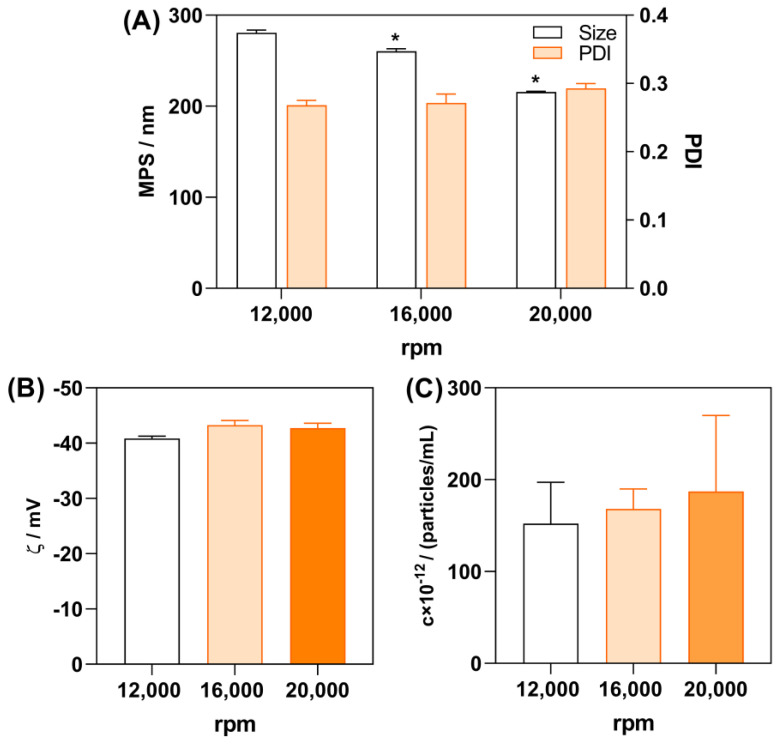
The effect of the homogenization rate on the mean particle size (MPS, **A**), polydispersity index (PDI, **A**), zeta potential (ζ, **B**), and particle concentration (**C**) of NLC_PS80 dispersion. One asterisk (*) denotes a statistically significant difference compared to the sample obtained at 12,000 rpm (*p* < 0.0001).

A further step in formulation development aimed to additionally reduce the NLC particle size by employing a higher homogenization rate (i.e., 16,000 rpm and 20,000 rpm; Figure 2). The elevated homogenization rate additionally reduced particle size and sample polydispersity index without significantly changing the zeta potential and concentration of the NLC_PS80 obtained (*p* > 0.05). When homogenization at 20,000 rpm was employed, NLC_PS80 of 215.5 ± 0.9 nm were obtained. 

It must be noted that during the NLC preparation, the PG was intentionally added to the lipid phase of the emulsion. The standard procedure employed in NLC preparation implies the addition of the preservative to the aqueous phase of the emulsion [13]. However, we have decided to proceed adversely, as we observed that PG addition to the lipid phase enhances the solubility of the components with high melting points (i.e., GMS and CH) in the melted mixture, where the PG acted as a co-solvent. Moreover, this protocol enabled the formation of smaller particles. When the PG was added to the aqueous phase, the same processing conditions led to the formation of larger particles with less homogenous particle size distribution (i.e., 325.5 ± 5.4 nm and PDI of 0.44 ± 0.2). However, the zeta potential of such a sample was −39.62 ± 0.95 mV, not significantly different from that obtained by the standard procedure (i.e., −42.72 ± 0.9, *p* > 0.05). The PG probably diffused to the aqueous phase of the formulation during the homogenization and particle solidification step. 

As microbial stability is one of the crucial attributes of every dermal product, we have tested the efficiency of the microbial preservation of the developed formulation. According to the data presented in Table 1, the developed NLC_PS80 dispersion complied with the requirements of the European Pharmacopeia for preparation for cutaneous application, confirming efficient preservation.

**Figure 3:**
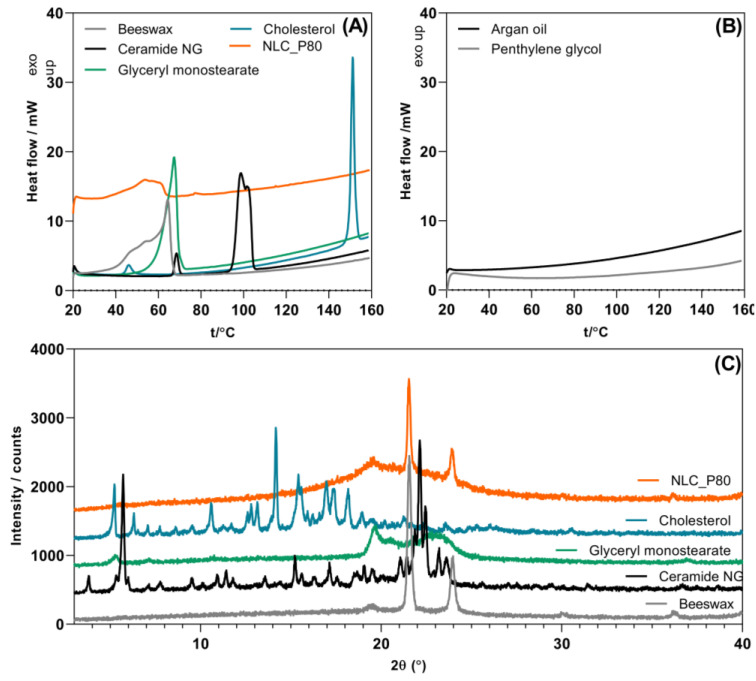
Solid-state analysis of lipid components and freeze-dried NLC_PS80 formulation performed by DSC (**A**,**B**) and XRPD (**C**).

To obtain information about the structural features of the NLC, the dispersions were freeze-dried and examined by DSC and XRPD analyses. The analysis of thermal events obtained by DSC provides information about the structure and physical state of the particles, as well as the interactions between components. The XRPD analysis is complementary to DSC, providing further insight into lipid polymorphism and NLC structure [10,12]. The DSC and XRPD results of NLC_PS80 are presented in Figure 3.

The BW showed a broad endothermic peak in the temperature range from 25 to 69 °C, peaking at 64.10 °C, representing the several polymorphic transitions of distinct components present in the material before melting. Depending on the geographic origin of the material, the melting point of BW can generally be observed between 61 and 67 °C [24]. Kameda and Tamada identified two polymorphic transitions occurring before the melting point of BW [25]. The first corresponds to the phase transformation from an orthorhombic to a rotator phase occurring at 36.4 °C. The second transition is attributed to a conformational transformation of the –CH_2_– chains from all-trans to a gauche–trans mixture with a corresponding DSC peak at 49.2 °C. Gaillard et al. [24] described an additional phase transition occurring at 55.2 °C, corresponding to the transformation between two rotator crystalline states (*rot. I* and *rot. II*, respectively).

The CH presented a first phase transition at 46 °C, typical for anhydrous pure cholesterol, followed by a second endothermic peak at 148.51 °C, corresponding to its melting point [26]. The characteristic split peak of the CE with an onset temperature of 91.47 °C indicates ceramide NG crystallized from methyl acetate. The exact thermal profile of CE is highly dependent on its thermal history and the heating rate employed during the DSC scan. Thus, the small exothermic peak at 68.53 °C may be attributed to the melting of its metastable form [27,28]. The GMS showed a broad endothermic peak ranging from 35.16–67.8 °C, probably caused by the simultaneous presence of both the β- and α-form GMS in the sample. The pure β-form shows a melting peak at 71.9 °C, while the pure α-form melts at 67.9 °C. Rapid cooling of melted GMS yields the α-form, which is successively transformed to the β-form via the β′-form under ambient conditions. Depending on the thermal history, the sample may contain a small amount of the α-form, decreasing the observed melting point [29]. The AO and PG presented no distinct thermal events in the studied temperature range.

The freeze-dried NLC_PS80 sample presented a broad exothermic peak from 35.16 to 65.50 °C, highly resembling that of the BW, the principal solid lipid comprised in the formulation. Compared to the BW, this peak was shifted to the lower temperatures due to the presence of the AO and other lipidic components in the NLC formulation [30]. This signal probably comprises similar complex polymorphic transitions occurring before melting to those of plain BW. Thus, the NLCs will be completely melted when the temperature exceeds 65.50 °C, while upon application to the skin (assuming skin temperature of 32 °C), they will remain solid [10]. 

To further elucidate the structure of the NLCs formed, an XRPD analysis was performed. The XRPD patterns shown in Figure 3C reveal the polycrystalline nature of the bulk lipids used in the formulation. BW is only partially crystalline, showing strong intensities reflections at 2θ of 21.58 and 23.98°, corresponding to the orthorhombic structure of the hydrocarbons/monoesters fractions [24]. The same signals were also observed for the NLC_PS80 sample, confirming that the BW retained its native structure inside NLC, as suggested by the DSC study. Their lower intensity may imply a decreased crystallinity and greater crystal defects of the BW when incorporated into NLC [31]. 

The GMS presented an XRPD diffraction pattern typical for β-form (Figure 3C), while a peak at 21.3°, typical of GMS α-form, was not observed [32]. However, its presence in the sample cannot be excluded. Yajima et al. demonstrated that the reflection typical of the α-form of GMS cannot be observed in the XRPD diffractogram when its content in the mixture is below 15% (*w*/*w*) [29]. Furthermore, the peak typical of GMS β-form at 19.5° may also be observed in the diffractogram of NLC_PS80, while typical peaks for other solid lipids employed in the formulation were absent. This indicates the formation of an imperfect crystal NLC type, typically occurring when mixing lipids with different chain lengths or using either mono-, di- or triglycerides [33]. BW and GMS were present in the most stable polymorphic forms in such a system, while CH and CE retained their amorphous nature. Using non-ionic surfactants with low melting points (i.e., −25 °C for PS80) favors the rapid conversion of the lipids to the most stable polymorphic form during NLC formation [12]. On the other hand, the amorphous state of the CH and CE might be attributed to their excellent solubility in the molten BW/AO mixture, facilitated by the PG acting as a co-solvent. Moreover, it appears that the GMS did not act solely as the emulsifier with PS80; it was also incorporated into the NLC structure as a structural solid lipid. The thermodynamic stability of lipid(s), their packing density, and polymorphic transitions during storage are critical for drug loading and release. In general, lipids present in their less stable crystalline forms (α and β′) tend to recrystallize to the most stable β-form during storage. This polymorphic transition from α to β′ and finally β form is responsible for the included drug expulsion. Also, the drug-loading capacity is related to the crystalline state of the lipids forming the NLC matrix [10]. 

Accelerated stability studies performed by centrifugation were aimed at estimating physical changes occurring during the storage of NLC_PS80, anticipating stability problems over time [34]. After each cycle, the MPS, PDI, and ZP of the samples were observed by DLS, and results are presented in Figure S1. The changes in the MPS, PDI, and ZP after each of the centrifugation cycles were statistically insignificant with respect to the initial value (*p* > 0.05), indicating the colloidal stability of the prepared NLC_PS80 dispersion. This was further confirmed by real-time stability studies showing no significant changes in the MPS and PDI of the NLC_PS80 sample after up to six months of storage, regardless of the conditions employed (Figure S2). Only a slight reduction in the ZP was observed during the storage time (*p* < 0.001), probably caused by subtle changes in pH due to CO_2_ dissolution in the aqueous phase of the NLC dispersion [35], which did not contain any buffer. However, this slight reduction in ZP did not lead to the physical instability of the NLC dispersion. 

### 2.2. Preparation and Characterization of NLC-Loaded Hydrogels

**Figure 4:**
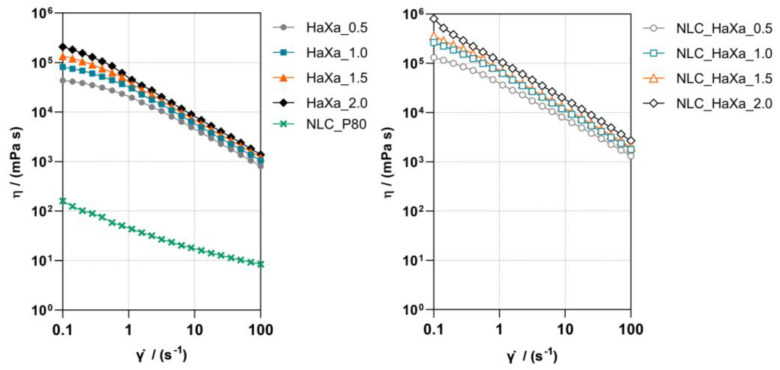
Shear viscosity profiles of blank (**left**) and NLC-loaded HaXa hydrogels (**right**) containing 1% (*w*/*w*) of HA and variable amounts of XA (0.5, 1.0, 1.5, and 2.0% (*w*/*w*), respectively). The shear viscosity curve of the optimized NLC dispersion (NLC_PS80) was added for comparison.

The low viscosity of the optimized NLC_PS80 dispersion (Figure 4) reduces skin retention of the formulation and makes it difficult to apply to the skin. The incorporation of NLC into semisolid bases like hydrogels is a suitable strategy to facilitate product application to the skin and increase product retention at the administration site [36]. The viscous property allows the hydrogel to flow and conform to the tissue defects and wounds, while the elastic property provides the hydrogel with the mechanical strength to resist deformation, thus prolonging the residence time [9]. Polysaccharide-based hydrogels are of particular interest as they are biocompatible and biodegradable, showing several positive effects on the skin and the skin pathologies, depending on the type of polysaccharide employed [9,37]. 

In this paper, the polysaccharides, HA and XA, were selected as the hydrogel components. HA is a negatively charged biodegradable polymer based on D-glucuronic acid and N-acetyl-D-glucosamine, forming weak hydrogels via hydrogen bonding and electrostatic interaction. Owing to their biocompatibility and functional versatility, those polysaccharides are widely employed in developing different advanced drug delivery systems [38,39,40,41,42,43]. In hydrogel development, the HA was employed at a 1% (*w*/*w*) concentration, typical for cosmetics [9,44]. The XA was also used in concentrations from 0.5 to 2.0% (*w*/*w*) to further enhance the viscosity of the hydrogels prepared [7].

The shear viscosity profile of the prepared blank and NLC-loaded HaXa hydrogels (Figure 4) revealed their shear thinning behavior, making them suitable for skin application. The shear created during the product application to the skin will cause a decrease in formulation viscosity, thus leading to excellent spreadability and patient compliance [45]. The increase in XA content dominantly contributed to the viscosity of blank hydrogels, and the addition of NLC_PS80 further increased the viscosity of NLC_HaXa hydrogels. The viscosities of NLC-loaded HaXa hydrogels were significantly higher than those of blank HaXa hydrogels, indicating that NLC_PS80 also contributed to the structure of the hydrogel formed. A similar result was observed upon incorporation of ibuprofen-loaded NLC into Carbopol^®^ 934 hydrogels [46]. Additionally, blank and NLC-loaded HaXa hydrogels presented thixotropy (Figure S3). The NLC_HaXa hydrogels presented a higher level of recovery of the viscosity 60 s after the strain was removed, ranging from 81.3% to 86.2% of the initial values. The recovery of blank HaXa hydrogels ranged from 78.7% to 80.0%. Transient and reversible interactions between the NLC surfaces and hydrophilic groups of the HA and XA polymers modulated the self-organization of hydrogels, thus promoting the recovery of their mechanical properties shortly after applying high shear stress [47]. Because of that, all NLC-loaded hydrogels can break down their structure facilitating the application followed by a fast restructuration after removing the strain, ensuring prolonged residence of NLC at the administration site. Furthermore, such reversible, isothermal, time-dependent shear thinning behavior of the prepared hydrogel systems is also crucial to obtain the desired spreadability and sensorial properties of the formulation [45,48]. 

**Figure 5:**
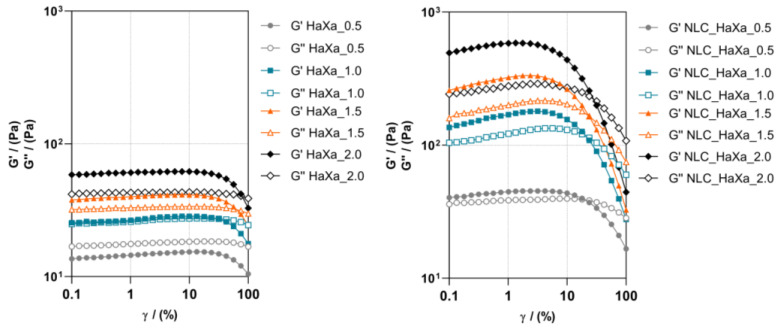
Dependence of storage (*G*′) and loss (*G*″) moduli on shear strain for blank (**left**) and NLC-loaded HaXa hydrogels (**right**) containing 1% (*w*/*w*) of HA and variable amounts of XA (0.5, 1.0, 1.5, and 2.0% (*w*/*w*), respectively).

For more comprehensive information on the rheological features of the prepared composite hydrogel formulations, oscillatory measurements were performed and the results of the amplitude sweep test are presented in Figure 5. 

In the linear viscoelastic region, for all samples except blank HaXa_0.5 hydrogel, the storage modulus (*G*′) values were higher than the loss modulus (*G*″) values, indicating the gel structure of the systems formed [31,49]. On the contrary, for blank HaXa_0.5 hydrogel, the *G*″ predominated the *G*′ values over the whole range of shear strains applied. It seems that 0.5% (*w*/*w*) of XA was not sufficient to form a gel with HA, resulting in a viscous liquid [49]. Higher XA concentrations (1.0, 1.5, and 2.0% (*w*/*w*)) lead to increased *G*′ and *G*″ values, resulting in stronger hydrogel formation. The addition of NLC_PS80 to the HaXa hydrogel further increased both *G*″ and *G*′ values, and when loaded with NLC_PS80, even the sample with 0.5% (*w*/*w*) of XA appeared as a gel. The improvement of hydrogel strength and viscoelasticity found in NLC_HaXa hydrogels may be attributed to the even distribution of NLC within the polymer matrix, which enabled enhanced hydrogen bonds/electrostatic interactions and consolidated the network structure [31]. Furthermore, while it is a highly hydrophilic material, HA also contains hydrophobic domains in the structure characteristic of lipids, thus promoting interaction with NLCs [44].

The values of tan *δ*, calculated from the *G*″/*G*′ ratio, represent the rate of dissipated and stored energy during a deformation cycle and indicate the gel strength. The gels with tan *δ* just below 1 are classified as weak; gel systems with values within the interval 0.1 < tan *δ* < 0.5 are considered to have medium strength, while values ≤ 0.1 indicate the formation of strong gels [50]. From the values of tan *δ* obtained at the angular frequency of 1 rad s^−1^ and deformation of 1% (Table S1), it is evident that all the hydrogel samples prepared can be classified as weak gels. The increase in XA content in the formulation and particularly the incorporation of the NLC_PS80 contributed to the strength of the formed hydrogel.

**Figure 6:**
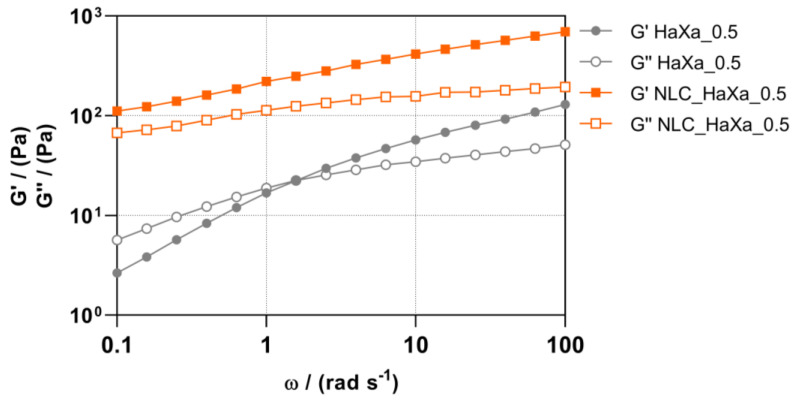
Storage (*G*′) and loss (*G*″) moduli as a function of angular frequency (ω) for blank and NLC-loaded HaXa hydrogel containing 1% (*w*/*w*) of HA and 0.5% (*w*/*w*) of XA.

As the HaXa hydrogel prepared with 1% (*w*/*w*) of HA and 0.5% of XA presented critical properties, it was selected for a frequency sweep test to study the angular frequency-dependent behavior of a sample in the non-destructive deformation range and to estimate its stability during storage. The results are presented in Figure 6. The blank HaXa_0.5 hydrogel presented gel-like properties (*G*′ > *G* ″) at higher frequencies, while at lower frequencies, it behaved as a viscous liquid (*G*″ > *G*′). The cross-over between the moduli appeared at 1.5 rad s^−1^. Such phase conversion indicates that the weak structure of the blank HaXa_0.5 hydrogel was destroyed and transformed into a liquid-like structure at low frequencies. This shows possible instability during the storage, as low frequencies simulate slow motion on long timescales or at rest [51]. For the NLC-loaded HaXa_0.5 hydrogel, the *G*′ modulus exceeded *G*″ in the whole range of examined angular frequencies, demonstrating the rheological stability of such hydrogel formulations [52].

**Figure 7:**
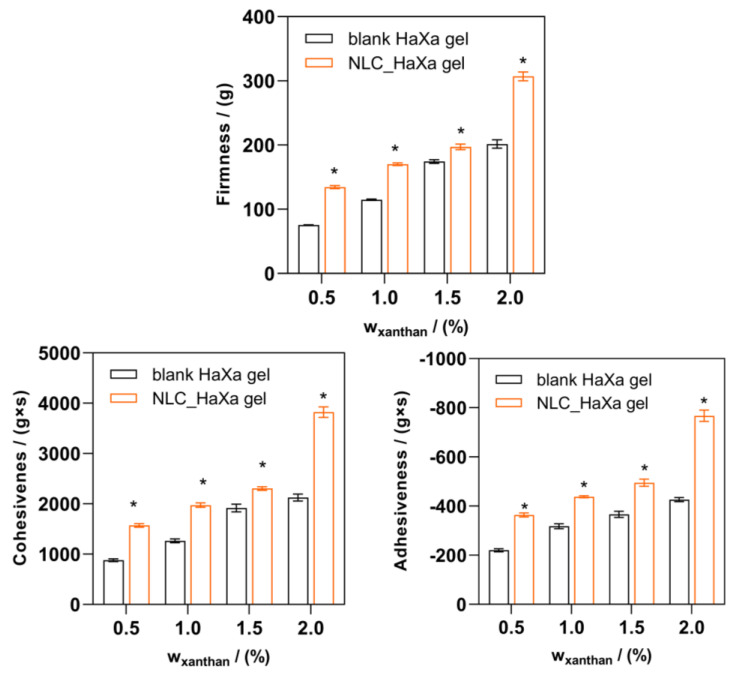
Textural properties of blank and NLC-loaded HaXa hydrogels containing 1% (*w*/*w*) of HA and variable amounts of XA (0.5, 1.0, 1.5, and 2.0% (*w*/*w*), respectively) obtained by the backward extrusion test. Asterisks (*) denote a statistically significant difference compared to the corresponding blank HaXa gel (*p* < 0.001).

The textural properties of the gel are an important parameter in the optimization of the topical formulation, affecting the sensorial properties during the product administration and governing its therapeutic outcome by providing close and prolonged contact between the NLC and the skin [53]. However, highly viscous hydrogels show high cohesiveness, so they are difficult to extract from the container and hard to apply to the skin, so the textural properties of the formulation must be finely tuned in the development phase [45,54]. The results of the textural analysis of blank and NLC-loaded HaXa hydrogels are presented in Figure 7.

The amount of the gelling agent in the formulation has a paramount influence on the textural properties of the formed hydrogel. In the case of the blank HaXa hydrogels, the firmness, cohesiveness, and adhesiveness increase linearly as a function of the polymer concentration, while in the case of the NLC-loaded HaXa hydrogel, the increase appears to be exponential. This observation further demonstrates the positive influence of NLC on the microstructure of the gel formed, observed by the viscoelastic rheological studies, contributing to the mechanical properties of such composite hydrogels. Some authors have also reported the positive effect of NLC loading on the textural properties of the hydrogels [31,55], while others reported the opposite effect, probably depending on the interactions occurring between the NLC and the gelling agent employed [56]. Regardless, both the hardness and consistency of the developed NLC_HaXa hydrogels are somewhat in between those of commercially available Neoprofen gel (Pensa, Turkey) and Ibuactive cream (Pharmactive Drug, Turkey), respectively, which were determined by Usta et al. under similar conditions [46]. This confirms the suitability of the developed NLC_HaXa hydrogels for dermal administration.

**Figure 8:**
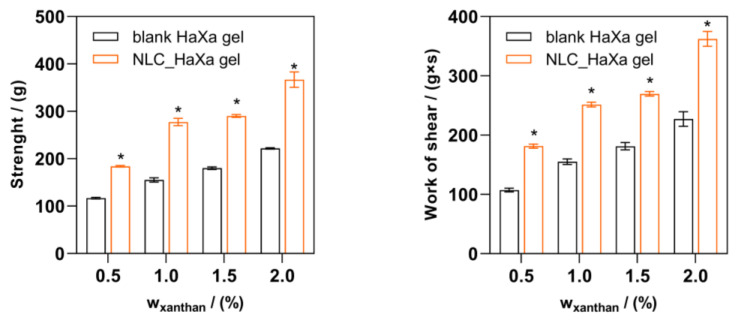
Spreadability parameters (strength and work of shear) of blank and NLC-loaded HaXa hydrogels containing 1% (*w*/*w*) of HA and variable amounts of XA (0.5, 1.0, 1.5, and 2.0% (*w*/*w*), respectively) obtained by the TTC spreadability test. Asterisks (*) denote a statistically significant difference compared to the corresponding blank HaXa hydrogel (*p* < 0.001).

Spreadability is among the essential properties of topical formulation that determine patient compliance. Adequate spreadability enables an even product application to the skin and coverage of larger skin areas during the application, improving the formulation’s effectiveness and enhancing patient compliance [57]. The spreadability of semisolid topical formulations is the net result of rheological contributions, of which viscosity is just one. In addition, structural and viscoelastic characteristics that describe the rigidity, strength, and relative contributions of elastic and shear thinning behavior play a major role in imparting spreading properties [58]. The developed NLC-loaded HaXa hydrogels showed low strength and spread at low shear, enabling comfortable gel application to the skin and thus ensuring patient compliance (Figure 8). The slight variation in the tested parameters is related to the XA concentration and loading of NLC, which determined the hydrogels’ rheological, viscoelastic, and textural properties, as previously discussed. 

The pH of blank HaXa hydrogels was around 6.5, independent of the XA concentration (Figure S4). Although NLC_PS80 dispersion showed a comparable pH to the blank hydrogels, their loading into the gels decreased the pH values to 5.8. The interaction of NLC with the polysaccharide compounds of the gels probably changed the dissociation state of d-glucuronic acids moieties of HA, changing the pH of the system formed. Regardless, the pH remained in the range acceptable for dermal application [59]. 

### 2.3. Biocompatibility of NLC-Loaded Hydrogel

**Figure 9:**
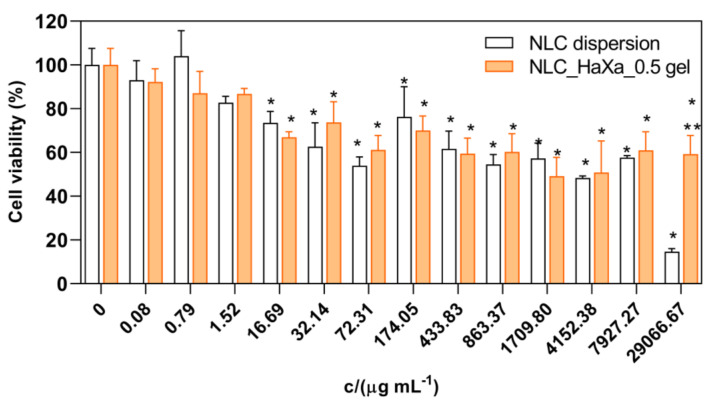
Biocompatibility of NLC_PS80 dispersion and NLC_HaXa_0.5 hydrogel on HaCaT cell line tested by MTT reduction assay. One asterisk (*) denotes a statistically significant difference compared to the negative control (*p* < 0.001), while two asterisks (**) denote a statistically significant difference compared to the NLC_PS80 dispersion at the same concentration (*p* < 0.001).

Evaluating biocompatibility is a mandatory quality aspect to address, even though most NLC-based formulations are composed of safe, well-known, and generally-recognized-as-safe components. The determination of cell viability by MTT assay is the most frequent test used to prove the biocompatibility of lipid nanoparticles [10,11]. In the case of topical NLC-based formulations, immortalized human epidermal keratinocyte cell line (HaCaT) is the most frequently employed, as keratinocytes represent the major cell type in the epidermis, and in vivo keratinocytes are biologically relevant targets for topically applied formulations [36,60,61]. The influence of NLC_PS80 dispersion and NLC_HaXa_0.5 hydrogel on the viability of the HaCaT cell line observed by MTT assay is presented in Figure 9.

The results revealed that cell viability of keratinocytes was dose-dependent, with a decrease when concentrations exceeded 16.69 μg mL^−1^ for NLC_PS80 dispersion and the NLC_HaXa_0.5 hydrogel, respectively. However, even at the highest concentration tested, the NLC_HaXa_0.5 gel demonstrated only a weak cytotoxic effect, with cell viability ranging from 60 to 80% [62]. It is important to note that during the in vitro experiments, the cell exposure to formulation is higher than that resulting after skin application of the formulation, as only a small portion of the NLC reaches the deeper epidermis, bypassing the *stratum corneum* and interacting with the viable keratinocytes [61,63]. Furthermore, the reinforcement and repair of the *stratum corneum* lipid film and increased skin hydration obtained by the protective lipid film formation on the skin upon dermal application of NLC can reduce or prevent skin irritation as well [11]. 

### 2.4. Functional Properties of NLC-Loaded Hydrogel

**Figure 10:**
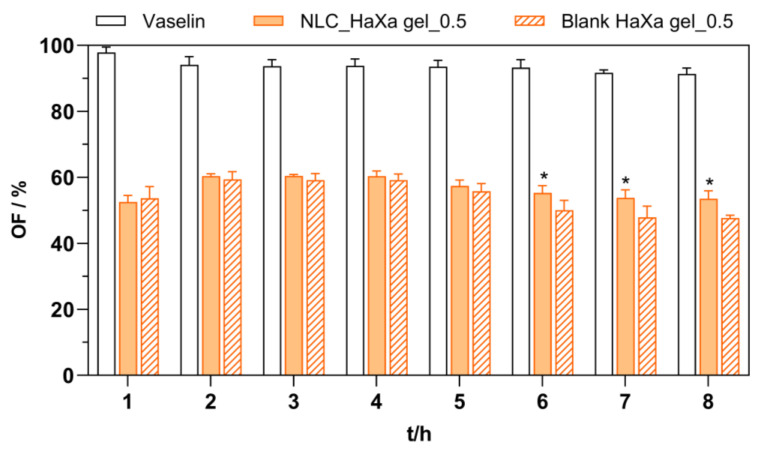
In vitro occlusion factor (OF) as a function of time for selected blank and NLC-loaded HaXa hydrogels containing 1% (*w*/*w*) of HA and 0.5% (*w*/*w*) of XA. Vaseline was used as a positive control. Asterisks (*) denote a statistically significant difference compared to the corresponding blank HaXa gel (*p* < 0.001).

The occlusive effect is a fundamental feature of topical formulation, supporting the physical skin barrier and enhancing the absorptive potential of the skin [3,10]. In this study, the occlusiveness of the blank and NLC-loaded HaXa gels was evaluated and compared to Vaseline, a well-known occlusive compound [64]. The representative results are given in Figure 10. Vaseline, as a positive control, presented almost complete occlusion in the in vitro model employed, presented nearly complete occlusion in the in vitro model used, which aligns with its well-documented clinical performance [64]. The occlusion factors obtained for both the blank and the NLC-loaded HaXa hydrogel containing 1% (*w*/*w*) of HA and 0.5% (*w*/*w*) of XA were significantly lower than those obtained for Vaseline (*p* < 0.05), reaching approximately 60% of that observed for Vaseline. Moreover, during the first 5 h of the study, the blank and NLC-loaded HaXa hydrogel presented comparable values. The statistically higher occlusiveness for the NLC-loaded HaXa hydrogel was observed after 6 h of the experiment. Initially, the high occlusiveness of the blank HaXa hydrogel may be attributed to HA, showing a high capacity to hold water [44], thus restricting the water permeation through the gel regardless of the presence of NLC in the formulation. However, after the formulation dried out, its occlusive effect decreased. At this point, the occlusive effect of the NLC became noticeable due to the formation of the coherent NLC (mono)layer at the surface of the membrane, further preventing water loss by evaporation [10]. Other hydrogel samples containing higher concentrations of XA presented a similar in vitro occlusion effect.

It was experimentally demonstrated that the occlusion factor of lipid nanoparticles with a size larger than 1 µm was only 10%, while an occlusion factor approaching 50% was obtained for NLCs of approximately 200 nm in size [65]. The size reduction in NLCs may further improve the occlusive effect of the NLC-loaded HaXa gels. Another aspect to consider is the crystallinity of lipids contained in the NLC. Wissing and Muller [66] have demonstrated that the occlusion factor depends strongly on the degree of crystallinity of the lipid matrix, i.e., this effect is proportional. For the non-crystalline formulation, an occlusive effect can hardly be detected after 6 h [66]. Finally, the concentration of NLC in the hydrogel may be another relevant factor, contributing to the formation of a more densely packed NLC layer on the skin surface, and, thus, enhancing the level of occlusion.

**Figure 11:**
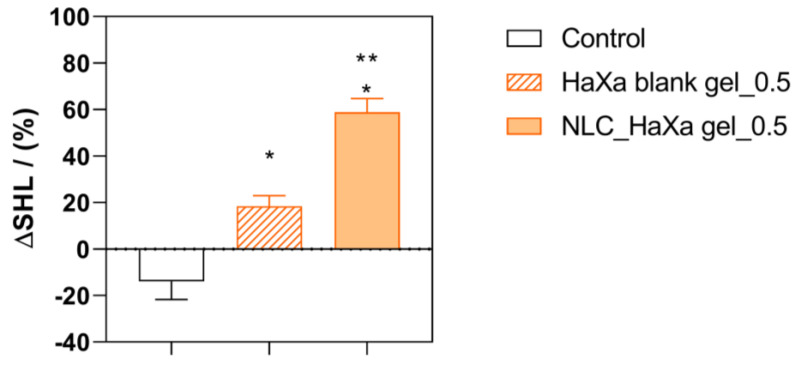
The relative changes in the skin hydration level (Δ*SHL*) observed after applying the blank and NLC-loaded HaXa_0.5 hydrogels on the ex vivo porcine ear model with disrupted epidermal barrier by SLS treatment. Untreated skin served as a control. One asterisk (*) denotes a statistically significant difference compared to the control (*p* < 0.001), while two asterisks (**) denote a statistically significant difference compared to the skin treated with blank HaXa_0.5 gel (*p* < 0.001).

The potential of the developed gel formulations to restore the epidermal skin barrier was assessed on an ex vivo porcine ear model. This model uses non-modified skin that is still connected to the cartilage of the tissue, thus maintaining highly physiological skin conditions [67]. The epidermal barrier was first disrupted by sodium lauryl sulfate (SLS), and its recovery was monitored by measuring the skin hydration level after applying the blank and NLC-loaded hydrogels. The hydration level of untreated skin was monitored as a reference. The results are presented in Figure 11.

The untreated (control) skin areas presented a reduction in the hydration level. As the excised porcine ear lacks active circulation, the tissue and the skin will invariably dehydrate throughout the experiment. This was further enhanced by the epidermal barrier impairment caused by the SLS treatment [68]. Despite that, treatment with blank HaXa_0.5 hydrogel increased the skin hydration level, probably due to the demonstrated occlusiveness of this formulation brought up by the HA and glycerol presence, which acted as a humectant [69]. The NLC-loaded HaXa_0.5 hydrogel showed superior efficiency by further enhancing the skin hydration level. This effect may be attributed to the AO and CE presence in the formulation, which alone showed an enhanced ability to hydrate the skin. Also, it is possible that the formed NLC_PS80 was incorporated into the intracellular lipid matrix of the skin, supplementing the lipid components extracted by the SLS treatment [1]. However, this assumption must be further examined, which is outside the scope of his study. Nonetheless, the results obtained agree with those of Tichota et al., which demonstrated that the increased skin hydration brought up by applying an NLC formulation with AO as a liquid lipid is a synergistic effect of NLC occlusion and argan oil hydration [2]. In the case of NLC_PS80, this effect was probably further promoted by CE’s ability to maintain and restore the epidermal skin barrier [3].

## 3. Conclusions

The developed nanostructured hydrogel formulation with functional NLC may be considered an alternative to commercially available, conventional dermocosmetic formulations used to repair the epidermal skin barrier, owing to the nanometric dimension and lipidic composition of NLCs. Among tested surfactants, the combination of PS80 and GMS enabled the preparation of NLCs of an imperfect crystal type with the targeted size range and acceptable colloidal stability. Incorporating the NLC-enhanced rheological, viscoelastic, and textural properties of the gels formed presented the suitable spreadability required for comfortable product application to the skin. The noticeable in vitro occlusion effect and efficient skin hydration observed in the ex vivo porcine ear model represented the functionality of formulation needed for treating skin with an impaired epidermal barrier function. However, further investigations are required in order to determine the exact clinical benefits of such nanocomposite formulations.

## 4. Materials and Methods

### 4.1. Materials

The argan oil (AO, CAS No. 223747-87-3), α-tocopheryl acetate (αTA, CAS No. 7695-91-2), beeswax (BW, CAS No. 31566-31-1), cholesterol (CH, CAS No. 57-88-5), glycerol (GLY, CAS No. 56-81-5), glyceryl monostearate (GMS; CAS No. 31566-31-1), Polysorbate 20 (PS20, CAS No. 9005-64-5), Polysorbate 60 (PS60, CAS No. 9005-67-8), Polysorbate 80 (PS80, CAS No. 9005-65-6), sodium hyaluronate (HA, CAS No. 9067-32-7, MW of 1.5–2.0 × 10^6^ Da), and xanthan gum (XG, CAS No. 11138-66-2) were obtained from Fagron, Zagreb, Croatia. The ceramide NG (CE) was purchased from Sederma, France, while the pentylene glycol (PG, CAS No. 5343-92-0) was purchased from Cosmaderm, Wuppertal, Tokyo, Germany. The sucrose stearate (SS, CAS No. 25168-73-4) and sucrose palmitate (SP, CAS No. 13039-41-3) were kindly donated by Mitsubishi-Kagaku Foods Corporation, Japan. The pluronic F-68 (F68, CAS No. 9003-11-6) and F-127 (F127, CAS No. 9003-11-6) were purchased from BASF SE, Rheinland-Pfalz, Germany. 

For the in vitro biocompatibility experiments, the HaCaT cell line (Cell Line Services, Eppelheim, Germany) was used. The chemicals used for cell culturing and experiments were Hank’s balanced salt solution (HBSS, pH 7,4), Dulbecco’s Modified Eagle Medium (DMEM with high glucose content; Sigma-Aldrich, Burlington, MA, USA), fetal bovine serum (FBS, Sigma-Aldrich, Burlington, MA, USA) penicillin, streptomycin and amphotericin B (1%; Lonza, Basel, Switzerland), and phosphate-buffered saline (PBS; Lonza, Switzerland). 

All other solvents and chemicals were of analytical grade and were used without further purification. 

### 4.2. Preparation of Functional NLC

**Figure 12:**
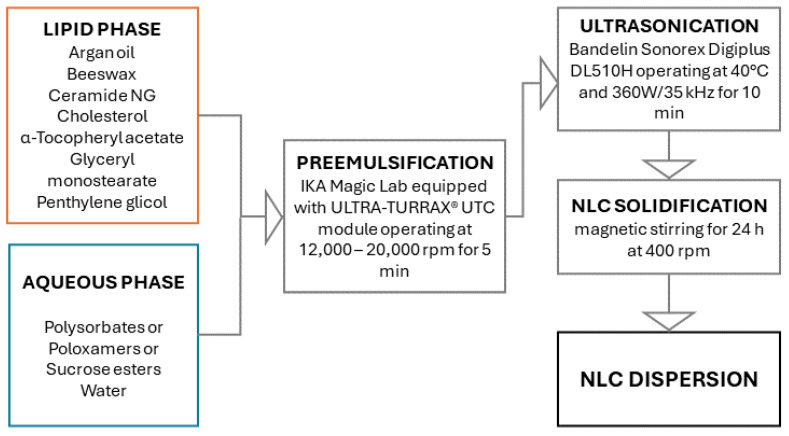
The preparation protocol of NLC dispersions.

The NLC preparation protocol is presented in Figure 12. The lipid phase containing 2.0 g of AO as liquid lipid and 4.26 g of BW, 0.60 g of CE, 0.30 g of CH, and 1.56 g of GMS as solid lipids was first heated to 80 °C in a thermostatic water bath to melt the solid components and then 0.02 g of αTA and 5.00 g of PG were added under moderate stirring to form clear oil phase. The preheated aqueous phase containing 84.2 g of purified water and 1.56 g of selected emulsifier was then added to the melted lipid phase. In this research, three different polysorbates (PS20, PS60, and PS80), two different sucrose ester derivatives (SS and SP) and two copolymeric surfactants (F68 and F127) were used as emulsifiers and the prepared formulations were named accordingly, stating the emulsifier type employed (i.e., NLC_PS20, NLC_PS60, NLC_PS80, NLC_SS, NLC_SP, NLC_F68 and NLC_F127, respectively). The primary emulsion was prepared by a high-shear mixer (IKA Magic Lab equipped with ULTRA-TURRAX^®^ UTC module) operating at different stirring rates ranging from 12,000 to 20,000 rpm for 5 min. The primary emulsion formed was then transferred to an ultrasonic water bath (Bandelin Sonorex Digiplus DL510H, BANDELIN electronic GmbH & Co., KG; Berlin, Germany) at 40 °C and treated with 360 W/35 kHz of ultrasound for 10 min, followed by magnetic stirring for 24 h at 400 rpm and ambient temperature to generate solid NLC. Three independent batches of each NLC formulation were prepared and characterized.

All samples prepared were stored in the refrigerator, protected from light until further use.

### 4.3. Characterization of Functional NLC

#### 4.3.1. Particle Size, Polydispersity Index, Zeta Potential and Concentration of NLC

The mean particle size (MPS) and polydispersity index (PDI) of the prepared NLC samples were determined at 25 °C by dynamic light scattering (DLS) using a Zetasizer Ultra (Malvern Panalytical Ltd., Malvern, UK) equipped with a He-Ne laser at a wavelength of 633 nm and a maximum power of 10 mW. Each NLC dispersion was diluted 50 times (*v*/*v*) using purified water. The material refractive index (RI) was set to 1.45 and its absorption to 0.01, while water was selected as the dispersant. Measurements were performed in triplicate at 25 °C with a 60 s equilibration time. The attenuation of the laser was determined automatically by the device to maintain the optimal count rate of scattered light while the angle of detection was set to 174.7°. 

The zeta potential (ZP) of the prepared NLC samples was determined using the same equipment and experimental setup. The electrophoretic light scattering was measured in triplicate at 25 °C with a 60 s equilibration time. The laser attenuation and voltage selection were set automatically. 

The particle concentration measurements using multi-angle dynamic light scattering (MALDS) were carried out using the same instrument and experimental settings. All MADLS measurements were collected at 3 different angles of detection, namely, backscatter (174.7°), side scatter (90°) and forward scatter (12.78°) with an equilibration time of 120 s. The instrument settings were optimized automatically. All data were presented as mean values ± SD of three independent measurements.

#### 4.3.2. Solid State Characterization of NLC

For solid-state characterization, optimized NLC dispersion was freeze-dried using Christ Epsilon 2-4 LSC Plus freeze drier. The freezing of the NLC dispersion in glass vials filled up to 1 cm height was performed at −40 °C for 2 h, followed by the primary drying at −20 °C and a vacuum of 0.5 mbar for 18 h. Secondary drying was performed at 4 °C and the vacuum of 0.05 mbar for 6 h. The solid product obtained was evaluated using differential scanning calorimetry (DSC) and X-ray powder diffraction (XRPD).

DSC analysis of the freeze-dried NLC formulation and starting components were performed using a Perkin-Elmer Diamond Differential Scanning Calorimeter (PerkinElmer, Inc., Waltham, MA, USA) calibrated with indium (99.98% purity; melting point 156.61 °C and fusion enthalpy of 28.71 Jg^−1^). Accurately weighted samples (2–5 mg, Mettler M3 Microbalance, Hessen, Germany) in sealed aluminum pans with pierced lids were scanned under the nitrogen purge (25 mL min^−1^) at a heating rate of 10 °C min^−1^ over the temperature range of 20–160 °C. 

Diffractograms of the starting compounds and freeze-dried NLC were recorded on a Malvern Panalytical Empyrean X-ray diffractometer in the 2θ range from 3° to 40°. An X-ray tube with a copper anode, X-ray output beam wavelengths λ(Kα1) = 1.54056 Å and λ(Kα2) = 1.54439 Å was the radiation source. The Kα1/Kα2 intensity ratio was 0.5, the working voltage of the tube was 45 kV, and the cathode was heated with a current of 40 mA.

#### 4.3.3. Stability Testing of NLC Dispersions

Accelerated stability testing was performed by centrifugation according to the protocol described by Gonçalves et al. [34]. The selected NLC dispersion was first diluted in purified water (1:100, *v*/*v*) and submitted to two 3000× *g* cycles (Thermo Scientific Heraeus Biofuge Stratos High-Speed Benchtop Centrifuge, Dreieich, Germany). After each cycle, samples were observed visually for the presence/absence of phase separation, creaming or flocculation, which predicted stability problems. The size, PDI and zeta potential were also evaluated as described above.

For the real-time stability studies, selected NLC dispersions were stored in sealed glass containers at 25 °C ± 2 °C/60% RH and 4 °C ± 2 °C/60% RH, respectively. At preselected periods (0, 30, 60, 90, 120, and 180 days), samples were withdrawn and analyzed for size, PDI and zeta potential as described above.

### 4.4. The Antimicrobial Preservative Efficiency Test

The antimicrobial preservative efficiency test was performed according to the monograph Ph. Eur. 10: 5.1.3. *Efficacy of antimicrobial preservation*. A series of NLC dispersions in sterile plastic containers were inoculated with one of the test organisms (*Staphylococcus aureus* ATTC 6538, *Escherichia coli* ATCC 8739, *Pseudomonas aeruginosa* ATCC 9027, *Candida albicans* ATCC 10231 and *Aspergillus brasiliensis* ATTC 16404) to give an inoculum of 10^5^ to 10^6^ microorganisms per gram of the preparation. The tested samples were stored at 22.5 ± 2.5 °C, protected from light. On days 2, 7, 14 and 28 following inoculation, 1 g of the inoculated sample was aseptically removed, adequately diluted, plated onto the agar medium used for the initial cultivation, incubated at prescribed conditions and then counted for viable microorganisms. 

### 4.5. Formulation of NLC-Loaded HaXa Hydrogels

The optimized NLC dispersion was incorporated into the hydrogel base consisting of sodium hyaluronate (HA, 0.5%, *w*/*w*) and xanthan gum (XA) at different concentration levels (i.e., 0.5, 1.0, 1.5, and 2.0%, *w*/*w*). The NLC-loaded hydrogels were prepared using the FagronLab™ PRO unguator (Bavaria, Germany). Half of the required amount of NLC dispersion was poured into the FagronLab™ vessel, followed by adding glycerol (10%, *w*/*w*) and the required amounts of HA and XA. Finally, the remaining amount of NLC dispersion was added. The mixing was performed for 26 min by applying 6 interchanging speed intervals (600 rpm vs. 1400 rpm) and times (485 s vs. 30 s vs. 510 s vs. 30 s vs. 485 s vs. 30 s).

Blank HaXa hydrogels were prepared following the same procedure but replacing the NLC dispersion with an aqueous solution containing 10% (*w*/*w*) of glycerol and 5% (*w*/*w*) of pentylene glycol. 

### 4.6. Rheological Characterization

Rheological measurements were performed using the Modular Compact Rheometer MCR102 (Anton Paar GmbH, Graz, Austria), which is equipped with a cone (1°) plate (diameter 50 mm, CP50) or parallel plate (diameter 50 mm, PP50) measuring system and an integrated air-cooled Peltier temperature control system. All data were calculated using the rheometer software RheoCompass^TM^ (Anton Paar GmbH, Austria). 

#### 4.6.1. Viscosity Curve

The viscosity curve was determined by a rotational test using the CP50-1 measuring system, with the gap set at 0.102 mm. All samples were equilibrated at 32 °C for 3 min. The viscosity profile was measured in the shear rate range of 0.1–100 s^−1^. Three replicate measurements were performed for each sample.

#### 4.6.2. 3-Interval Thixotropy Test

A 3-Interval Thixotropy Test (3-ITT) was performed to test the thixotropy properties of the developed gels. The test was conducted at 32 ºC using the CP50-1 measuring system. The gap was set at 0.102 mm. In the first interval, the viscosity of the formulation was measured at the shear rate of 1 s^−1^ for 10 s. During the second interval, a shear rate of 1000 s^−1^ was applied to the formulation for 5 s. The third interval showed the viscosity of the formulation at the starting shear rate, i.e., at 1 s^−1^ in a duration of 65 s. All samples were measured in triplicate.

#### 4.6.3. Amplitude Sweep Test

An amplitude sweep test of gels was performed employing a parallel plate measuring system (PP50), with the gap set at 0.500 mm. The samples were equilibrated at 32 °C for 3 min. Storage (*G*′) and loss (*G*″) modulus were measured at the constant angular frequency of 1 rad s^−1^ and within the amplitude range from 0,1 to 100%. The measurement was performed in triplicate.

#### 4.6.4. Frequency Sweep Test

A PP50 measuring system was used for the frequency sweep test with the gap set at 0.500 mm. The samples were kept on the lower measuring plate at 32 °C for 10 min before measurement. The dependence of storage (*G*′) and loss (*G*″) modulus on angular frequency in the range from 0.1 to 100 rad s^−1^ was measured at the amplitude 1% (from linear viscoelastic range). 

### 4.7. Textural Analysis

A texture analyzer TA.XT Plus (Stable Micro Systems Ltd., Godalming, UK), equipped with a 5 kg load cell, was used to study the texture properties and spreadability of the hydrogels prepared. The measurements were performed at ambient temperature.

Textural analysis was performed using a back extrusion rig with a diameter of 40 mm. Approximately 75 g of the hydrogel formulation was filled into a standard back extrusion container with a diameter of 50 mm, avoiding the introduction of air bubbles. The back extrusion rig was then immersed into the hydrogel to a depth of 20 mm at a velocity of 2 mm s^−1^ and withdrawn, applying the return velocity of 20 mm s^−1^. Three separate measurements were performed for each formulation. The texture parameters of the hydrogels were determined from the resultant force-time curve. The maximum compression force (in g) presented the hardness of the gel. Cohesiveness was defined as the work required to deform the gel in the down movement of the probe and was calculated from the resultant area under the force-time curve. The second area under the force obtained during the probe retraction represented the adhesiveness of the hydrogel [53]. 

The spreadability of the hydrogels was measured using the TTC spreadability fixture. During the test, the conical probe was lowered over a distance of 23 mm at a speed of 5 mm/s to the surface of the hydrogel in the support, then penetrated the sample, forcing it to flow outwards at 45° between the surfaces of the two cones (male and female). The force (in g) required to penetrate the male-type conical probe into the sample from the female-type conical support was measured. From the force-time curve, two parameters were selected as indicators of the strength and spreadability of the hydrogels: the maximum force value and the work of shear, respectively. The work of shear was determined from the area under the curve in the region with positive force values [54]. Three separate replicates were performed for each formulation.

### 4.8. pH Measurements

The pH of both blank and NLC-loaded HaXa hydrogels was recorded using a Mettler S47 SevenMulti™ dual pH/conductivity meter equipped with a pH InLab^®^ Viscous electrode (Mettler-Toledo, Greifensee, Switzerland). The measurements were performed in triplicate on undiluted hydrogel samples. The Mettler pH Sensor InLab^®^ Expert Pro-ISM electrode was employed to measure the pH of NLC dispersion. Here, the measurements were also performed in triplicate on undiluted samples.

### 4.9. Cell Culture and Biocompatibility Test

#### 4.9.1. Cell Culture Conditions

The human keratinocyte cell line HaCaT was cultured in DMEM supplemented with 10% FBS and 1% penicillin, streptomycin and amphotericin B. The cell medium was changed every 2 days. The cells were passaged at 80–90% confluence. The cells were kept in the incubator (Heracell VIOS 160i CO_2_ Incubator, ThermoFisher Scientific, Waltham, MA, USA) at 37 °C, 95% humidity, and in a 5% CO_2_ atmosphere. The cells were detached from the flasks by soaking first in EDTA solution and then with trypsin/EDTA (0.25%/0.02% in phosphate-buffered saline PBS, respectively). The cells were split in ratios from 1:5 to 1:10. The passages used for biocompatibility studies were 8–10. 

#### 4.9.2. In Vitro Cell Biocompatibility Study

In vitro biocompatibility of the leading NLC_HaXa_0.5 hydrogel and corresponding controls was determined by 3-(4,5-dimethylthiazol-2-yl)-2,5-diphenyltetrazolium bromide (MTT) assay. The cells were seeded in 96-well plates at a density of 10^4^ cells per well. After 48 h, the cells were washed with HBSS and treated with formulations mixed within volume ratios from 1:2 to 1:100,000 for 2 h at 37 °C. Pure HBSS was used as a negative control. Each formulation was tested in quadruplicate. Afterwards, the formulations were removed from the wells, the cells were rinsed with HBSS and incubated with the cell medium. Cell viability was determined after 24 h by MTT colorimetric assay. MTT was dissolved in sterile PBS (2.5 mg ml^−1^) and diluted by DMEM to the final concentration of 0.5 mg mL^−1^. The cells were exposed to MTT reagent (100 μL per well) for 45 min, after which the reagent was removed, the cells were lysed, and the formazan crystals were dissolved in 100 μL (per well) of acid isopropanol (4% 1 M HCl in isopropanol; *v*/*v*). The amount of formazan was determined spectrophotometrically by SpectraMax i3x multi-mode microplate reader (Molecular Devices LLC, San Hose, CA, USA) at the wavelength of 570 nm. Cell viability was calculated with respect to control (cells incubated in HBSS) using the following expression (Equation (1)):

(1) $$Cell\;viability\;\left(\%\right)=\frac{A_{sample}-A_{ipr}}{A_{control}-A_{ipr}}\times 100,$$

where *A_sample_* is the absorbance of formazan solution from the cells treated with tested formulations, *A_control_* is the absorbance of formazan solution from untreated cells (incubated with HBSS), and *A_ipr_* is the absorbance of pure acid isopropanol at the same wavelength.

### 4.10. Functional Characterization of the Developed HaXa Hydrogels 

#### 4.10.1. In Vitro Occlusion Test

An in vitro occlusion test was performed using PTFE immersion cells with adjustable volume and exposure area of 4 cm^2^ (Agilent Technologies, Santa Clara, CA, USA). The reservoir of the immersion cells was filled with 1 mL of purified water and covered with a membrane (regenerated cellulose filters, 0.2 µm pore size, Sartorius, Göttingen, Germany). The cell was then assembled, 1 g of the tested sample was applied onto the membrane and evenly spread using a spatula, forming a thin layer at the surface. The cells were incubated in an ICH110eco Climate Chamber (Memmert GmbH, Schwabach, Germany) set to 32 °C and 50% RH to simulate the conditions present at the skin surface. At the predetermined time points, the residual amount of water in the reservoir of the cells was measured gravimetrically (Mettler Toledo ML204T analytical balance, Hessen, Germany), to assess the water loss resulting from the evaporation through the membrane/gel layer. For each sample, experiments were performed in triplicate. The cells without the applied hydrogel sample were used as a negative control, while Vaseline was used as a positive control. The occlusion factor (OF) for each time point was calculated according to the following equation:

(2) $$OF=100\times \frac{A-B}{A}$$

where *A* and *B* are the observed water loss values for the cells without and with the sample applied, respectively. An occlusion factor of zero indicated no occlusive effect compared with the reference, and 100 was the maximum occlusion factor [70].

#### 4.10.2. Ex Vivo Skin Hydration Test

The fresh porcine ears were obtained from a local slaughterhouse and used within 2 h after slaughter. First, the ears were washed with lukewarm water and then carefully dabbed with a soft paper tissue. Intact skin areas (2.5 × 2.5 cm) without visible wounds or scratches were selected and marked. Hair within these areas was carefully cut short to 1 mm with a clipper. The skin samples were then thermostated at 32 °C and 50% RH for 30 min. After that, the initial values of the skin moisture were recorded using a Multi Skin Test Center 1000 equipped with a probe measuring the skin moisture based on capacitance (Courage + Khazaka Electronic GmbH, Köln, Germany). Three independent measurements were taken at different positions for each selected field to avoid false readings due to skin maceration. Then, the integrity of the epidermal barrier was disrupted using the modified procedure described by Čuříková-Kindlová et al. [5]. Briefly, each of the selected areas was treated by placing the paper tissue soaked with 10% sodium lauryl sulfate (SLS) solution for 8 h. Petri dishes with the treated pig ears were covered with a parafilm sealing film to avoid evaporation and thermostated at 32 °C and 50% RH. After that, the SLS-soaked paper tissues were removed, and the skin was thoroughly washed and dried using a soft paper tissue. A total of 100 µL of each formulation (i.e., HaXa_0.5 and NLC_HaXa_0.5 hydrogel) was applied on three different skin areas of three independent ears and evenly spread using a spatula. One of the marked areas on each ear was left untreated, serving as a control. After the product application, ears were again thermostated at 32 °C and 50% RH for 1 h, and then the skin hydration levels were measured again, as described above. Before the measurement, the eventual product residue was carefully removed with soft tissue paper [71]. The relative change in the skin hydration level (Δ*SHL*) was calculated according to the following formula:

(3) $$∆SHL=100\times \frac{S{LH}_{1}-{SHL}_{0}}{{SHL}_{0}}$$

*SHL*_0_ and *SHL*_1_ denote measured skin hydration levels before disruption of the epidermal barrier and 1 h after formulation administration to the skin, respectively [72]. 

### 4.11. Statistical Evaluation

All results are expressed as mean ± standard deviation (SD) of N separate measurements. The statistical calculations were performed using GraphPad Prism 8 for Windows (GraphPad Software, Boston, MA, USA). A one-way ANOVA was used to compare the means of different groups with one independent variable, followed by Dunett’s multiple comparisons test. To compare the means of different groups with two independent variables, a two-way ANOVA was employed, followed by Tukey’s multiple comparisons test. A value of *p* ≤ 0.05 was considered statistically significant.

## Figures and Tables

**Table 1:** The efficiency of antimicrobial preservation of the formulation NLC_PS80.

Test Microorganisms	Initial Concentration log_10_ cfu/mL	Log_10_ Reduction			
		2 Days	7 Days	14 Days	28 Days
*Staphilococcus aureus* ATTC 6538	5.66	4.66	4.66	4.66	Ø
		(2) *	(3) *	(3) *	(NI) *
*Escherichia coli* ATCC 8739	5.89	4.89	4.89	4.89	Ø
		(2) *	(3) *	(3) *	(NI) *
*Pseudomonas aeruginosa* ATCC 9027	5.95	4.95	4.95	4.95	Ø
		(2) *	(3) *	(3) *	(NI) *
*Candida albicans* ATCC 10231	5.91	4.91	4.91	4.91	Ø
		(2) *	(3) *	(3) *	(NI) *
*Aspergillus brasiliensis*	5.58	4.58	4.58	4.58	Ø
ATTC 16404		(-) *	(-) *	(2) *	(NI) *

*—required reduction in the count of viable microorganisms according to the monograph 5.1.3. of the Ph. Eur. 11.0; NI—no increase; Ø—not detected.

## Data Availability

The original contributions presented in the study are included in the article/Supplementary Material further inquiries can be directed to the corresponding author/s.

## Supplementary Materials

The following supporting information can be downloaded at: https://www.mdpi.com/article/10.3390/gels10070466/s1, Figure S1: Accelerated stability test of the optimized NLC_PS80 dispersion: mean spherical diameter (*MSD*, A), polydispersity index (*PDI*, B) and zeta potential (*ζ*, C) of the dispersion immediately after preparation (0), one and two cycles of centrifugation at 3000× *g* for 30 min (1 and 2, respectively). Before the experiments, NLC_PS80 dispersion was diluted with purified water 100× and all measurements were performed in triplicate; Figure S2: Real-time stability studies of the optimized NLC_PS80 dispersion stored at 25 and 4 °C, respectively, monitoring the mean spherical diameter (MSD, A), polydispersity index (PDI, B), and zeta potential (ζ, C) as a function of time. One asterisk (*) denotes a statistically significant difference compared to the initial sample stored at the same temperature (*p* < 0.0001), while two asterisks (**) denotes a statistically significant difference compared to the sample in the same time point stored at 25 °C (*p* < 0.0001); Figure S3: Time-dependent viscosity change in blank (left) and NLC-loaded HaXa hydrogels containing different amounts of XA (0.5, 1.0, 1.5, and 2.0% (*w*/*w*), respectively) as observed by 3-interval thixotropy test; Figure S4: pH of blank (left) and NLC-loaded HaXa hydrogels containing different amounts of XA (0.5, 1.0, 1.5, and 2.0% (*w*/*w*), respectively). Asterisk (*) denotes a statistically significant difference compared to the corresponding blank HaXa hydrogel (*p* < 0.0001); Table S1: Influence of xanthan content and NLC loading to tan *δ* values for the HaXa hydrogels at 32 °C. The values presented were obtained at the angular frequency of 1 rad s^−1^ and deformation of 1%.
