# Supplementary material for: Functional Nanostructured Lipid Carrier-Enriched Hydrogels Tailored to Repair Damaged Epidermal Barrier
Source: Gels. 2024 Jul 16;10(7):466. doi: 10.3390/gels10070466 (PMC11275585; doi:10.3390/gels10070466)
Supplement: Supplementary file 1 [file gels-10-00466-s001.zip › gels-3098794-supplementary.pdf]

Electronic Supplementary Information (ESI)

Functional Nanostructured Lipid Carrier-Enriched Hydrogels Tailored to Repair Damaged Epidermal Barrier

Radwan Joukhadar, Laura Nižić Nodilo, Jasmina Lovrić, Anita Hafner, Ivan Pepić and Mario Jug *

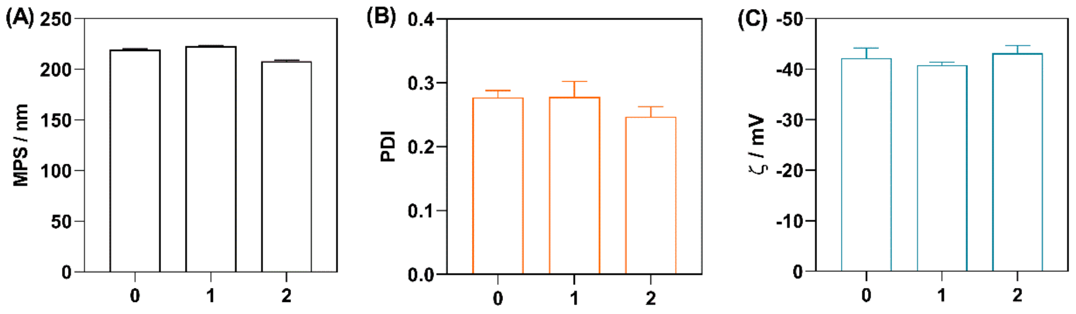

Figure S1. Accelerated stability test of the optimized NLC_PS80 dispersion: mean particle size (*MPS*, A), polydispersity index (*PDI*, B) and zeta potential (ζ, C) of the dispersion immediately after preparation (0), one and two cycles of centrifugation at 3000 × *g* for 30 min (1 and 2, respectively). Before the experiments, NLC_PS80 dispersion was diluted with purified water 100× and all measurements were performed in triplicate.

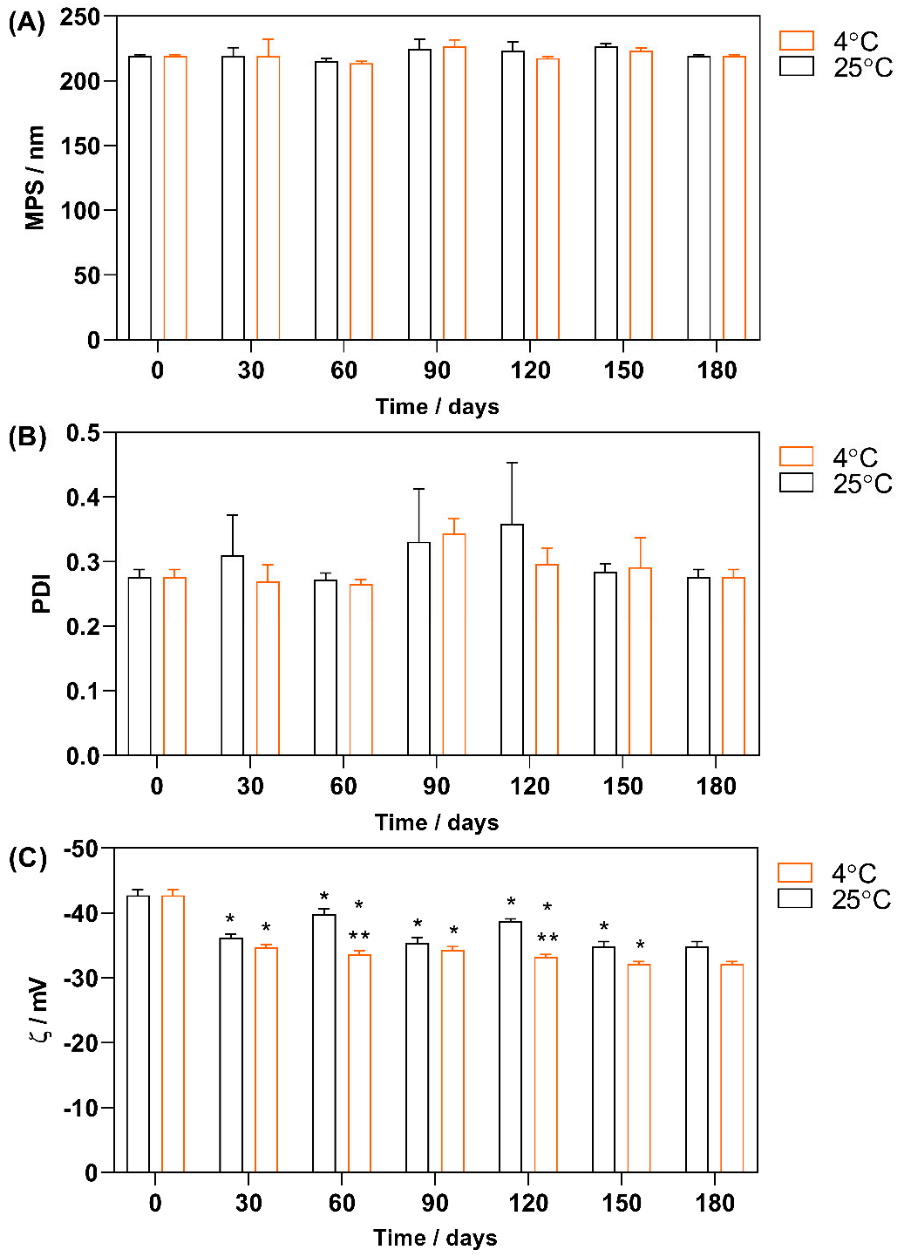

Figure S2. Real-time stability studies of the optimized NLC_PS80 dispersion stored at 25 and 4°C, respectively, monitoring mean particle size (*MPS*, A), polydispersity index (*PDI*, B), and zeta potential (ζ, C) as a function of time. One asterisk (*) denotes a statistically significant difference compared to the initial sample stored at the same temperature ($p < 0.0001$), while two asterisks (**) denotes a statistically significant difference compared to the sample in the same timepoint stored at 25°C ($p < 0.0001$).

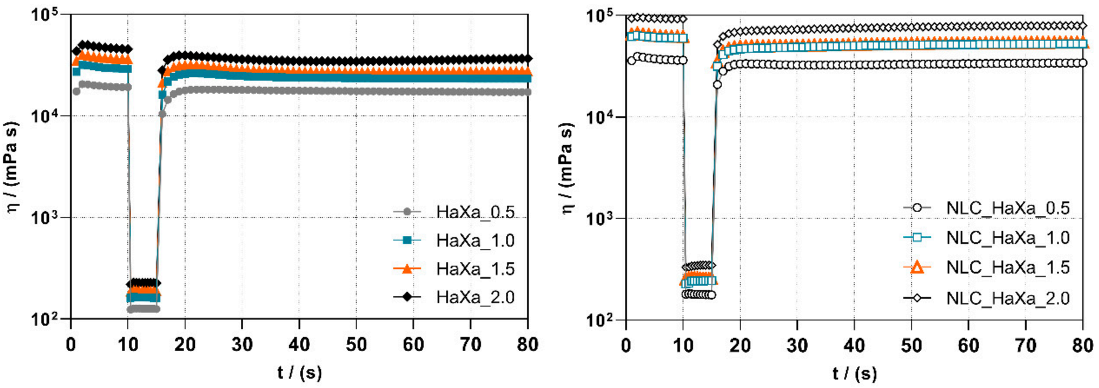

Figure S3. Time-dependent viscosity change of blank (left) and NLC-loaded (right) HaXa hydrogels containing different amounts of XA (0.5, 1.0, 1.5 and 2.0% (*w*/*w*), respectively) as observed by 3-interval thixotropy test.

Table S1. Influence of xanthan content and NLC loading to tan *δ* values for the HaXa hydrogels at 32°C. The values presented were obtained at the angular frequency of 1 rad s^{-1} and deformation of 1%. Asterisk (*) denotes a statistically significant difference compared to the corresponding blank HaXa hydrogel ($p < 0.0001$).

xanthan / (%)	tan *δ*	
	HaXa hydrogel	NLC_HaXa hydrogel
0.5	1.214 ± 0.015	0.874 ± 0.014 *
1.0	0.973 ± 0.009	0.734 ±0.024 *
1.5	0.827 ± 0.010	0.635 ± 0.031 *
2.0	0.709 ± 0.007	0.490 ± 0.030 *

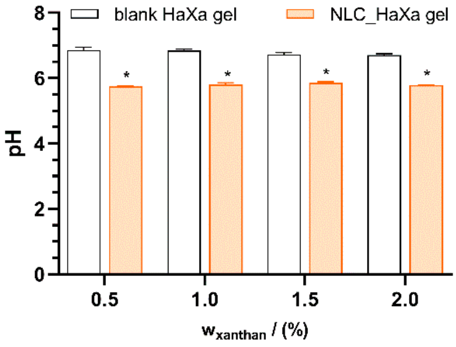

Figure S4. The pH of blank (left) and NLC-loaded (right) HaXa hydrogels containing different amounts of XA (0.5, 1.0, 1.5 and 2.0% (*w*/*w*), respectively). Asterisk (*) denotes a statistically significant difference compared to the corresponding blank HaXa hydrogel ($p < 0.0001$).
